# Poor sleep quality is a predictor of severe hypoglycemia during comprehensive diabetes care in type 1 diabetes

**DOI:** 10.1007/s11325-025-03385-1

**Published:** 2025-06-13

**Authors:** Prapai Dejkhamron, Thipaporn Tharavanij, Supawadee Likitmaskul, Petch Rawdaree, Jeerunda Santiprabhob, Chaicharn Deerochanawong, Wannee Nitiyanant, Sirimon Reutrakul, on behalf of Thai-Type 1 Diabetes and Diabetes diagnosed Age before 30 years Registry, Care and Network (T1DDAR CN) committee and members

**Affiliations:** 1 Department of Pediatrics, and Northern Diabetes Center, Faculty of Medicine, Chiang Mai University, Chiang Mai, Thailand; 2 Department of Medicine, Faculty of Medicine, Center of Excellence in Applied Epidemiology, Center of Excellence in Nexus for Advanced Intelligence in Law, Engineering, and Medicine (Nail’Em), Thammasat University and Thammasat Diabetes Center of Excellence, Thammasat University Hospital, Pathumthani, Thailand; 3 Siriraj Diabetes Center of Excellence and Department of Pediatrics, Faculty of Medicine Siriraj Hospital, Mahidol University, Bangkok, Thailand; 4 Department of Medicine, Faculty of Medicine, Vajira Hospital, Navamindradhiraj University, Bangkok, Thailand; 5 Rajavithi Hospital and Department of Medicine, College of Medicine, Rangsit University, Pathumthani, Thailand; 6 Siriraj Diabetes Center of Excellence and Department of Medicine, Faculty of Medicine, Siriraj Hospital, Mahidol University, Bangkok, Thailand; 7 Division of Endocrinology and Metabolism, Department of Medicine, Faculty of Medicine Ramathibodi Hospital, Mahidol University, Bangkok, Thailand; 8 Division of Endocrinology, Diabetes and Metabolism, Department of Medicine, University of Illinois Chicago, 835 S. Wolcott, Suite E625, Chicago, IL 60612 USA

**Keywords:** Sleep quality, Hypoglycemia, Type 1 diabetes

## Abstract

**Purpose:**

Sleep disturbances is common in type 1 diabetes (T1D) and can be associated with poor glycemic control, and possibly hypoglycemia. This study aims to investigate whether poor sleep quality, as assessed by the Pittsburgh Sleep Quality Index (PSQI), was associated with glycemic control or severe hypoglycemia in T1D individuals.

**Methods:**

This one-year prospective cohort study included 221 (148 F/63 M) T1D participants (aged ≥ 13 years), receiving intensive insulin therapy. A1C levels were obtained at baseline and during the 12-month follow-up. Incidences of diabetic ketoacidosis (DKA) and severe hypoglycemia were collected.

**Results:**

The mean age of participants was 21.4 ± 8.9 years, with a baseline A1C of 9.27 ± 2.61%. Poor sleep quality was reported in 33.0% of participants. A1C levels improved over the one-year follow-up, but there was no significant difference in A1C reduction between those with good vs. poor sleep quality (-0.42 ± 1.73 vs. -0.42 ± 1.67, *P* = 0.835), nor in DKA incidence (*P* = 0.466). However, participants with poor sleep quality experienced more SH episodes (6.53 (2.45–17.41) vs. 0 per 100-person year, *P* = 0.01). After adjusting for age, body mass index, and glucose monitoring, each one-point increase in PSQI score was associated with a higher severe hypoglycemia risk (OR 1.31, 95%CI 1.13–1.52). Poor sleep quality predicted an increased risk of severe hypoglycemia (OR 24.54, 95%CI 1.31-459.29).

**Conclusion:**

Poor sleep quality is common in T1D individuals and is a risk factor for incident SH. These findings highlight the importance of incorporating sleep assessment into routine T1D diabetes care and the need of targeted interventions to improve sleep quality in T1D individuals. These findings support the importance of addressing sleep quality in T1D management, particularly in relation to hypoglycemia risk.

## Introduction

Sleep is emerging as an important contributor to health in people with diabetes [1]. Sleep disturbances (e.g. poor quality, insufficient duration) have been found to impact glycemic control and psychological wellbeing [2]. People with type 1 diabetes (T1D) commonly experience sleep disturbances. For example, self-reported poor sleep quality was found in two-thirds of older adults [3] and 26% of adolescents [4]. These sleep disturbances have been shown to be associated with poor glycemic control [5]. The relationship between sleep and glycemic control in T1D is complex and likely bi-directional [6]. For example, sleep disturbances have been link to increased insulin resistance [7] and poor diabetes self-managements [8], possibly leading to hyperglycemia. Hyperglycemia, in turn, can negatively affect sleep causing increased arousals [9] and poor sleep quality [10]. Besides hyperglycemia, hypoglycemia warrants special consideration. Hypoglycemia, particularly nocturnal hypoglycemia, is common in T1D and can also disrupt sleep quality, leading to next-day fatigue and decreased overall wellbeing. In addition, the use of diabetes technologies has shown mixed effects on sleep [6]. Currently, the American Diabetes Association recommends evaluating sleep as a part of a comprehensive care in people with diabetes [1].

It is known that the incidence of T1D has increased worldwide, with a current estimate of 15 per 100,000 people and a prevalence of 9.5% [11]. Achieving good glycemic control remains the most crucial factor related to diabetic complications [1]. The availability of advanced diabetes technologies such as continuous glucose monitoring (CGM), automated insulin delivery (AID) and the use of insulin analogues have been shown to improve glycemic control and reduce hypoglycemia [12]. However, these modern treatments remain inaccessible in limited resource settings. For example, in a survey of five countries in Southeast Asia (Laos, Malaysia, Vietnam, Cambodia and Myanmar), no countries covered glucose monitoring [13]. Not surprisingly, the mean hemoglobin A1C (A1C) in these countries was 9.7%, compared to 7.7% reported in the T1D exchange cohort who were using CGM in the US [12, 13]. A similar trend was observed in Thailand, where glucose test strips and CGM were not covered by the national healthcare plan, in a study of 1,892 T1D patients revealed an average A1C of 9.35% [14]. In addition, diabetes self-management education and support (DSMES), which has been shown to improve A1C and self-care behaviors, is often not structured for T1D nor reimbursed in these countries [15]. To address these challenges, initiatives have been taken in these limited resource settings. In Thailand, the Thai Type 1 Diabetes and Diabetes Diagnosed Before Age 30 Years Registry, Care and Network (T1DDAR CN) was established in 2014 as a collaborative effort to strengthen T1D care through referral networks, structured DSMES, along with providing intensive insulin therapy (IIT) and glucose monitoring supplies (test strips and glucometers). These initiatives have demonstrated improvements in glycemic control. As a result, Thailand’s National Health Security Office (NHSO) expand treatment coverage and train diabetes care teams, providing nationwide support for individuals with T1D [16].

Most of the studies exploring sleep in people with T1D have been cross-sectional in nature and conducted in the Western countries [5], with only a few focused on Asian populations. Two small studies in Thailand revealed that approximately 40% of T1D participants reported poor sleep quality, with a mean sleep duration of less than 6.5 h/night [17, 18]. Given that most studies have been cross-sectional in design, it remains unclear whether sleep could longitudinally affect diabetes outcomes. Therefore, the aims of this study were to explore sleep quality of T1D patients in Thailand who were enrolled in TIDDAR CN, describe their characteristics associated with poor sleep quality and determine whether sleep was a predictor of metabolic control during a comprehensive care for T1D.

## Materials and methods

This one-year prospective cohort study is part of the Thai Type 1 Diabetes and Diabetes diagnosed Age before 30 years Registry, Care and Network (T1DDAR CN), which established the Diabetes Self-Management Program and Network System (DSMP-NS). This program has been implemented across 37 hospitals in Thailand since February 2019. Detailed descriptions of the T1DDAR CN and DSMP-NS have been published previously [16].

In this analysis, we included adolescents and young adults with T1D (aged 13 years and older) who self-reported their sleep quality at baseline. All participants completed least six essential DSMES modules covering topics such as general knowledge, diabetes complications, insulin treatment, glycemic monitoring, carbohydrate counting, and sick day care. Additional DSMES modules on exercise and social activities, special situations, pregnancy, and psychosocial issues were provided as appropriate. This training was provided at enrollment and at least once annually thereafter. All participants used IIT, either multiple daily injections (MDI; ≥4 injections/day) or continuous subcutaneous insulin infusion (CSII). They were provided free-of-charge blood glucose monitoring (BGM) devices, glucose test strips, and urine ketone strips. The program emphasized individualized insulin therapy using insulin analogs, with adjustments based on carbohydrate intake and blood glucose levels. Participants were advised to perform BGM at least three times daily, and a referral system within the T1DDAR CN network facilitated access to specialized care when needed.

### Baseline assessments and self-reported sleep quality

At baseline, data were collected on demographic information (age, duration of diabetes, weight, height, body mass index [BMI]), glycemic control status, frequency of self-monitoring blood glucose (SMBG), and chronic diabetes complications (such as diabetic retinopathy, nephropathy, and neuropathy). Glycemic control was categorized as follows (1) *good glycemic control*: A1C less than 7.5% for individuals under 18 years, and less than 7.0% for those aged 18 years or older; (2) *fair glycemic control*: A1C between 7.5% and 9.0% for individuals under 18 years, and between 7.0% and 9.0% for those aged 18 years or older; and, (3) *poor glycemic control*: A1C greater than 9% for all age groups. Diabetic retinopathy was defined as the presence of macular edema, proliferative or nonproliferative retinopathy, vitreous hemorrhage, or tractional retinal detachment. Diabetic nephropathy was diagnosed based on persistent albuminuria (≥ 30 mg/gram creatinine) in at least 2 out of 3 tests. Diabetic neuropathy was identified through monofilament examination, loss of reflexes or loss of vibratory sensation. Obesity was defined as a BMI of ≥ 25 kg/m^2^ for individuals aged 18 years or older, or a BMI > + 2 standard deviation score (SDS) for those under 18 years.

Self-reported sleep quality was obtained at baseline using the Thai version of the Pittsburgh Sleep Quality Index [19]. PSQI scores ranged from 0 to 21, with higher scores indicating poorer sleep quality. A cutoff score of greater than 5 indicates poor sleep quality.

### Assessments during follow up

During the one-year follow up, data on self-care practices, SMBG frequencies, routine physical examinations (body weight, height), A1C levels, and of acute diabetes complications were collected at 3 and 6 months (± 45 days), and 12 months (± 90 days). The occurrences of DKA and severe hypoglycemia (defined as hypoglycemia requiring assistance) were retrieved from medical records or self-reported when medical records were unavailable. SMBG frequencies were extracted from blood glucose meter downloads.

To ensure data accuracy, two auditors reviewed the data for all participants. Any discrepancies were resolved through verification with the relevant sites until consensus was reached. This study adhered to the Strengthening the Reporting of Observational Studies in Epidemiology (STROBE) guidelines for cohort studies.

This study complied with the Declaration of Helsinki and received approval from the Central Research Ethics Committee CREC021/61BRm) and by the institutional review board of each participating center. Informed consent/assent was obtained from all participants or the parents/legal guardians.

### Statistical analysis

Data analysis was conducted using Stata/IC version 14.0 for Windows (StataCorp LP, College Station, TX, USA). Categorical variables are presented as frequencies and percentages, while continuous variables are reported as means ± standard deviations for normally distributed data, or as medians with interquartile ranges (IQR) for non-normally distributed data. Group comparisons were performed using the Student’s t-test or Mann–Whitney U test for continuous variables, as appropriate, and the Chi-square test for categorical variables. Repeated measures analysis was used to assess differences in A1C levels at baseline, during treatment, and one year post-treatment between participants with poor and good sleep quality. Logistic regression analysis was conducted to identify independent predictors of hypoglycemia. Potential covariates, including age, diabetes duration, gender, BMI, and SMBG frequency, were first analyzed using univariate analysis. Variables found to be significant were subsequently included in a multivariable Firth logistic regression model. To address missing data, including those related to diabetes duration and complications, multiple imputation was performed. The results from the imputed datasets were compared with those obtained using pairwise deletion, and the findings were consistent. A *P*-value of less that 0.05 was considered statistically significant.

## Results

### Baseline clinical characteristics, sleep quality and metabolic control during follow-up

A total of 221 T1D participants (148 F/63 M) were enrolled, with 119 participants aged 18 years or older (Table 1). The overall median duration of diabetes was 8.60 years (IQR: 7.94-9.26years). Baseline A1C was 9.27 ± 2.61%, with 18.72% of participants achieving glycemic targets. All participants were on IIT: 220 using multiple daily injections (≥ 4 injections/day) and 1 using CSII. A total of 192 participants completed the 1-year follow-up. As compared to children/adolescents, adult participants had better glycemic control but a higher prevalence of diabetic retinopathy and nephropathy. Overall, 33.03% of participants (*n* = 73) reported poor sleep quality, with a median PSQI score of 4 (IQR: 3–6). Adult participants reported poorer sleep quality than children/adolescents (*P* < 0.01).

**Table 1:** Baseline characteristics of participants and metabolic control during follow up

	Total	Children and adolescent	Adults	*P*-value
		Age 13 to < 18 years	Age ≥ 18 years	
	(221 cases)	(102 cases)	(119 cases)	
Gender (female), n(%)	148 (66.97)	64 (62.75)	84 (70.59)	0.217
Age (years)	21.40 ± 8.97	15.44 ± 1.47	26.50 ± 9.55	< 0.001
Diabetes duration (years), mean (95% CI)	8.60 (7.94, 9.26)	5.56 (4.89, 6.24)	11.20 (10.12, 12.28)	< 0.001^*^
BMI SDS score (Age 13 to < 18 years)	0.25 ± 1.08	0.25 ± 1.08	-	-
BMI for adults (kg/m^2^)	22.91 ± 3.62	-	22.91 ± 3.62	-
Obesity at enrollment, n (%)	39/218 (17.89)	6/101 (5.94)	33/117 (28.21)	< 0.001
A1C (baseline), %	9.27 ± 2.61	9.96 ± 2.88	8.66 ± 2.19	< 0.001
Glycemic control at baseline				
Good glycemic control, n (%)	41 (18.72)	20 (19.61)	21 (17.95)	0.001
Fair glycemic control, n (%)	87 (39.73)	28 (27.45)	59 (50.43)	
Poor glycemic control, n (%)	91 (41.55)	54 (52.94)	37 (31.62)	
Diabetes retinopathy, % (95% CI)	7.73 (4.33, 11.14)	0.09 (-0.81, 1.01)	14.28 (7.99, 20.57)	< 0.001
Diabetes nephropathy, % (95% CI)	15.93 (11.18, 20.67)	8.04 (2.69, 13.38)	22.69 (15.16, 30.21)	0.002
Diabetic peripheral neuropathy, % (95% CI)	2.31 (0.33, 4.29)	0.09 (-0.81, 1.01)	4.20 (0.60, 7.81)	0.030
PSQI score	4 (3–6)	4 (2–5)	5 (3–7)	0.001^*^
Poor sleep PSQI score > 5, n (%)	73/221 (33.03)	23/102 (22.55)	50/119 (42.02)	0.002
***Metabolic control and SMBG frequencies during follow-up***				
Average A1C during 12-month	9.02 ± 2.29	9.63 ± 2.46	8.49 ± 1.99	< 0.001
Glycemic control in 1 year				
Good glycemic control, n (%) Fair glycemic control, n (%) Poor glycemic control, n (%)	44 (19.91) 90 (40.72) 87 (39.37)	21 (20.59) 31 (30.39) 50 (49.02)	23 (19.33) 59 (49.58) 37 (31.09)	0.009
DKA incidence (per 100 person- Year)	3.51 (1.58–7.82)	4.87 (1.83–12.98)	2.26 (0.56–9.02)	0.401
Severe hypoglycemia (per 100 person- year)	2.05 (0.77–5.47)	2.14 (0.53–8.54)	1.97 (0.49–7.89)	0.941
SMBG frequencies SMBG 0–1 time/days SMBG 2 time/days SMBG 3 time/days SMBG ≥ 4 time/days	2.75 (2–3.5) 54 (24.43) 58 (26.24) 70 (31.67) 39 (17.65)	3 (2–3.67) 25 (24.51) 23 (22.55) 32 (31.37) 22 (21.57)	2.75 (2–3.5) 29 (24.37) 35 (29.41) 38 (31.93) 17 (14.29)	0.534^*^ 0.450

^*^Two-sample Wilcoxon rank-sum (Mann-Whitney) test

Good glycemic control: A1C < 7% in adults or < 7.5% in children/adolescents

Fair glycemic control: A1C 7–9% adults or 7.5-9% in children/adolescents

Poor glycemic control: A1C > 9%

Data are presented as numbers and percentages or medians with interquartile ranges (IQR), unless otherwise noted.

Abbreviations: BMI, body mass index; SDS, standard deviation score; DKA, diabetic ketoacidosis; PSQI, Pittsburgh Sleep Quality Index; SBMG, self-monitoring of blood glucose

During the 1-year follow-up, the average A1C was 9.02 ± 2.29%, with adults maintaining better glycemic control compared to children/adolescents. The median frequency of SMBG was 2.75 times/day. The incidence of DKA incidence was 3.51 per 100 person-year, while the incidence of severe hypoglycemia was 2.05 per 100 person-year, with no significant differences between adults and children/adolescents.

### Comparison of baseline and follow up clinical characteristic between good and poor sleep quality groups

Participants with poor sleep quality were significantly older than those with good sleep quality (24.13 ± 10.07 vs. 20.05 ± 8.07 years, *P* = 0.003). However, there were no significant differences between the two groups in terms of gender distribution, diabetes duration, BMI, or the presence of diabetes-related complications between the two groups. Baseline glycemic control was also comparable.

**Fig. 1:**
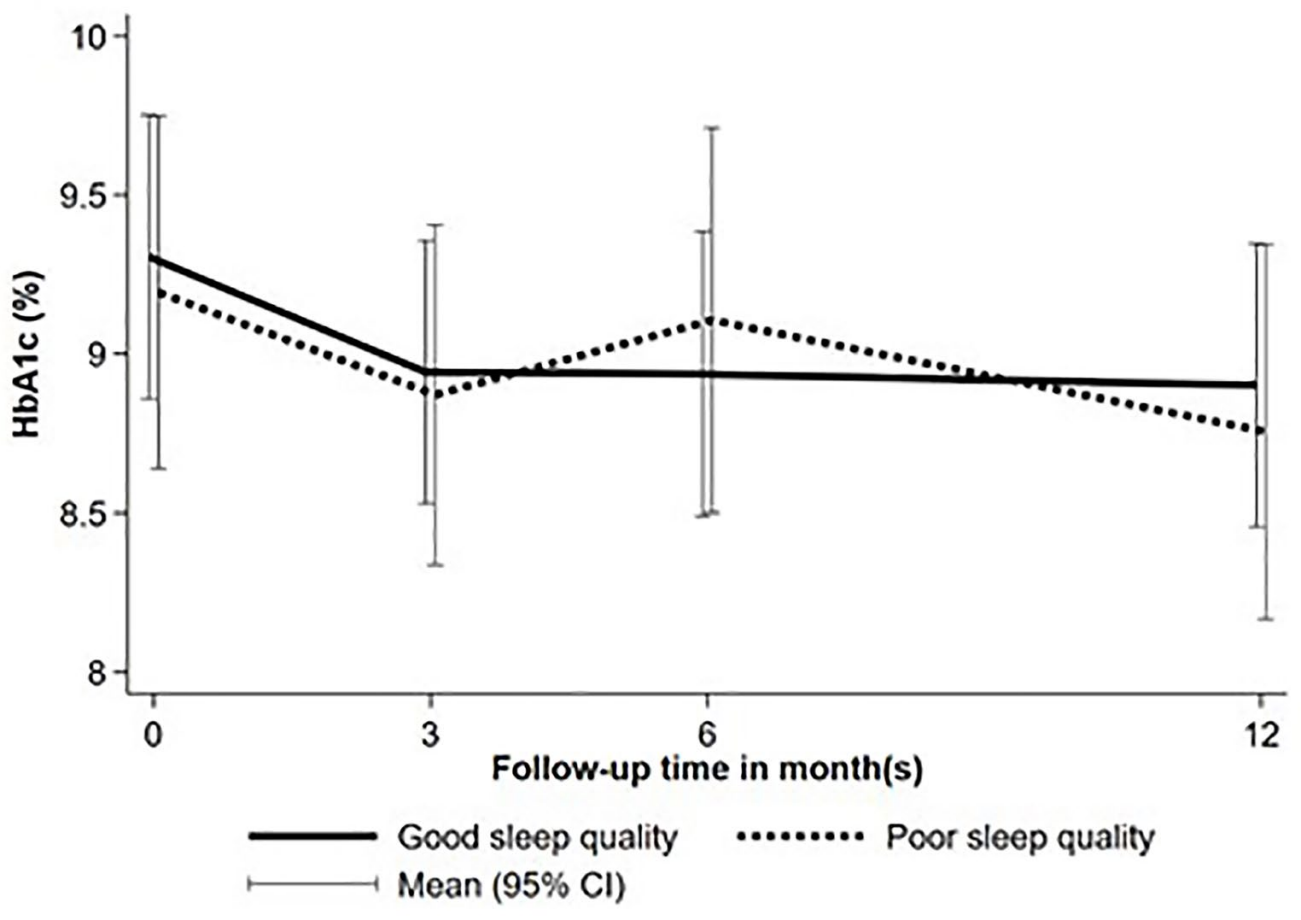
A1C at baseline and during 12-month follow up

**Fig. 2:**
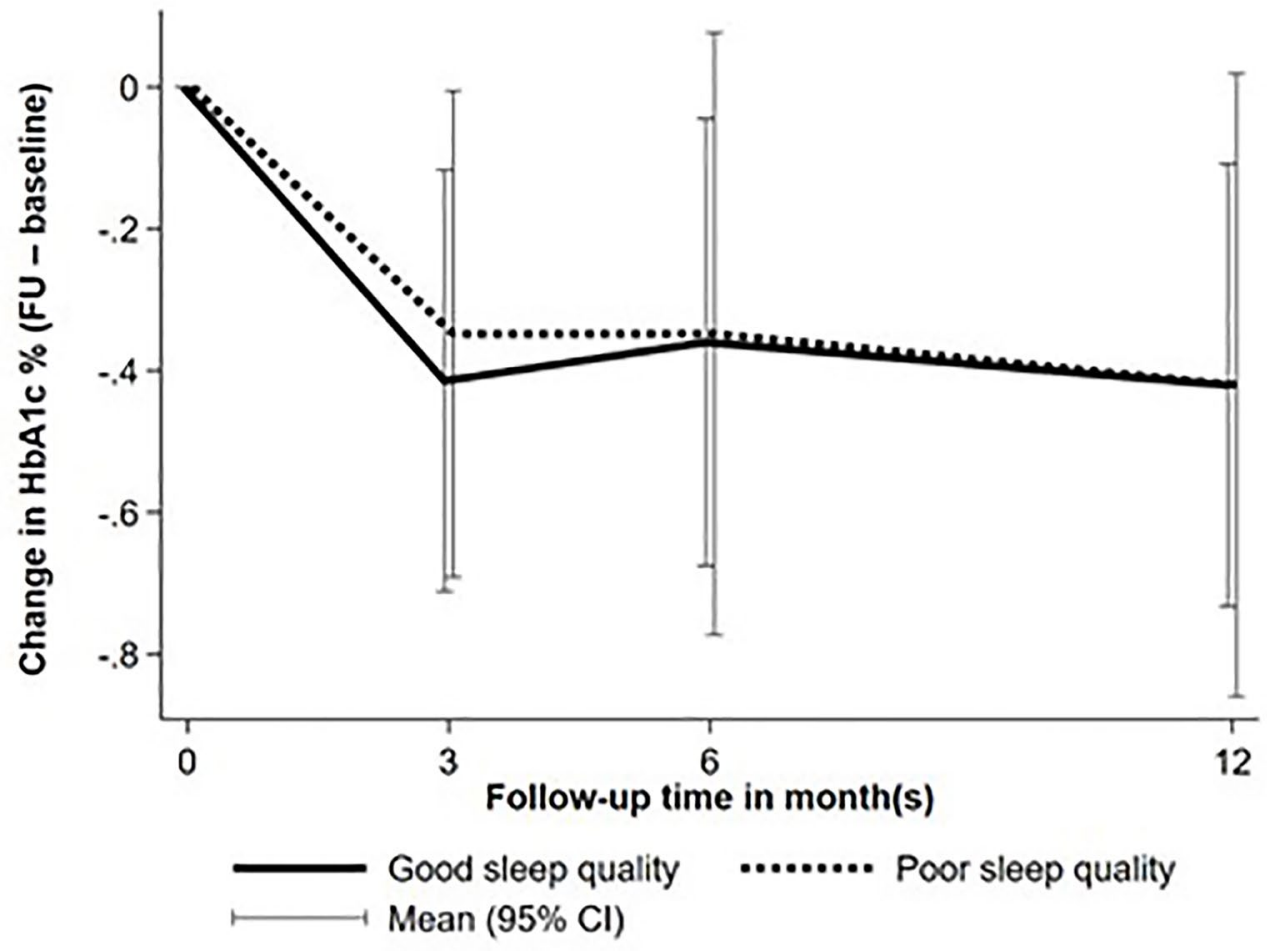
Changes in A1C at baseline and during 12-month follow up

Throughout the follow-up period, there were no significant differences between participants with good and poor sleep quality in average A1C levels at 3, 6, and 12 months, the proportion achieving glycemic targets (Table 2; Fig. 1), or changes in A1C over time (Fig. 2). The frequency of SMBG, as well as the proportions of participants with diabetic retinopathy, neuropathy, or the incidence of DKA, were also comparable between the two groups. However, participants with poor sleep quality experienced significantly more severe hypoglycemic events compared to those with good sleep quality (6.53 vs. 0 events per 100 person-years, *P* < 0.001, Table 2).

**Table 2:** Characteristic of patients with good compared to poor self-reported sleep quality

	Poor sleep quality	Good sleep quality	*P*-value
	(73 cases)	(148 cases)	
Gender (female), n (%)	48 (65.75)	100 (67.57)	0.787
Age (years)	24.13 ± 10.07	20.05 ± 8.07	0.003
Diabetes duration (years), mean (95% CI)	8.99 (7.70, 10.26)	8.33 (7.39, 9.27)	0.417
BMI for adults (kg/m2)	22.25 ± 3.14	23.39 ± 3.89	0.094
BMI Z score (Age 13 to < 18 years)	0.36 (-0.78–0.93)	0.34 (-0.28–0.94)	0.546^*^
Obesity at enrollment, n (%)	11/72 (15.28)	28/146 (19.18)	0.480
Hypertension, n (%)	0/73 (0.00)	8/148 (5.41)	0.055
A1C (baseline),%	9.19 ± 2.36	9.31 ± 2.74	0.768
Glycemic control at baseline			
Good glycemic control, n (%)	14 (19.44)	27 (18.37)	0.895
Fair glycemic control, n (%)	27 (37.50)	60 (40.82)	
Poor glycemic control, n (%)	31 (43.06)	60 (40.82)	
Average PSQI score	7 (6–9)	3 (2–4)	< 0.001^*^
Diabetes retinopathy, % (95% CI)	11.23 (3.89, 18.57)	6.15 (2.25, 10.04)	0.231
Diabetes nephropathy, (% (95% CI)	13.70 (5.81, 21.59)	17.03 (10.94, 23.11)	0.513
Diabetic peripheral neuropathy, % (95% CI)	4.38 (-0.46, 9.23)	1.49 (-0.55, 3.52)	0.283
***Metabolic control and SMBG frequencies during follow-up***			
Average A1C in 1 year	8.97 ± 2.07	9.04 ± 2.39	0.839
Glycemic control			
Good glycemic control, n (%) Fair glycemic control, n (%) Poor glycemic control, n (%)	11 (15.07) 33 (45.21) 29 (39.73)	33 (22.30) 57 (38.51) 58 (39.19)	0.402
Change in A1C% from baseline to 12-month	-0.42 ± 1.67	-0.42 ± 1.73	0.835^†^
DKA incidence (per 100 person- year)	5.04 (1.62–15.62)	2.70 (0.87–8.36)	0.466
Severe hypoglycemia incidence (per 100 person- year)	6.53 (2.45–17.41)	0	0.010
SMBG frequencies per day	2.5 (1.75–3.5)	3 (2–3.5)	0.170^*^

^*^ Two-sample Wilcoxon rank-sum (Mann-Whitney) test

^†^ Repeated measures mixed model

PSQI > 5 indicated poor sleep quality

Data are presented as numbers and percentages or medians with interquartile ranges (IQR), unless otherwise noted.

Abbreviations: BMI, body mass index; SDS, standard deviation score; DKA, diabetic ketoacidosis; PSQI, Pittsburgh Sleep Quality Index; SBMG, self-monitoring of blood glucose

### Sleep quality as a predictor of severe hypoglycemia

To explore if sleep quality was an independent predictor of severe hypoglycemia during follow-up, logistic regression analysis was conducted adjusted for age, BMI, and SMBG frequency. The results identified that each point increase in PSQI score was associated with an increased risk of severe hypoglycemia with an adjusted odds ratio of 1.311 (95% CI 1.129–1.523, *P* < 0.001). (Table 3). Greater SMBG frequency was also associated with lower risk of severe hypoglycemia (*P* = 0.024). In addition, participants with poor sleep quality (PSQI > 5) were 24.31 times more likely to experience severe hypoglycemia during a follow-up than those with good sleep quality (Table 4).

**Table 3:** Sleep quality score as a predictor of severe hypoglycemia during follow up

	Severe hypoglycemia						
Characteristics	Univariate analysis			Logistic regression			
	OR	95% CI	*P*-value	AOR	95% CI	*P*-value	
Age (years)	0.963	0.900–1.030	0.268	0.922	0.834–1.019	0.110	
BMI (baseline) kg/m^2^	1.082	0.847–1.381	0.528	1.084	0.849–1.383	0.517	
SMBG frequencies per day	0.362	0.167–0.789	0.011	0.300	0.106–0.855	0.024	
PSQI score	1.224	1.106–1.354	0.000	1.311	1.129–1.523	0.000	

Abbreviations: BMI, body mass index; SDS, standard deviation score; PSQI, Pittsburgh Sleep Quality Index; SBMG, self-monitoring of blood glucose

**Table 4:** Poor sleep quality as a predictor of severe hypoglycemia during follow up

Characteristics	Severe hypoglycemia						
	Univariate analysis			Firth logistic regression			
	OR	95% CI	*P*-value	AOR	95% CI	*P*-value	
Age (years)	0.963	0.900–1.030	0.268	0.944	0.812–1.098	0.454	
BMI (baseline) kg/m^2^	1.082	0.850–1.378	0.521	1.076	0.854–1.355	0.536	
SMBG frequencies							
SMBG 0–1 time/day	Ref.^*^						
SMBG 2 time/day	0.651	0.123–3.446	0.614	0.503	0.089–2.847	0.437	
SMBG 3 time/day	0.104	0.005–2.065	0.138	0.124	0.006–2.585	0.178	
SMBG ≥ 4 time/day	0.186	0.009–3.711	0.271	0.249	0.011–5.482	0.378	
Poor sleep quality	23.847^*^	1.300–437.390	0.033	24.310	1.30–455.26	0.033	

^*^ Firth Logistic Regression

Abbreviations: BMI, body mass index; SDS, standard deviation score; PSQI, Pittsburgh Sleep Quality Index; SBMG, self-monitoring of blood glucose

**Fig. 3:**
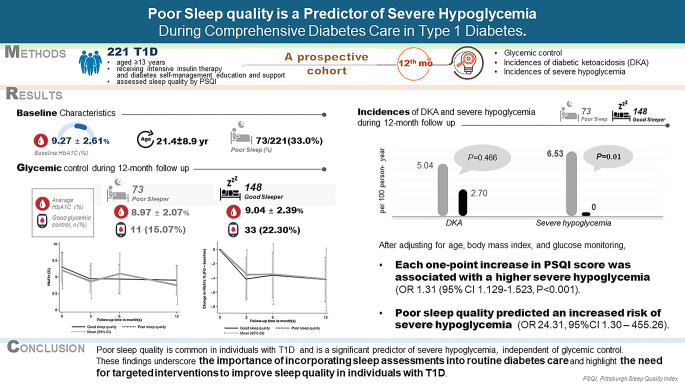
Summary of this prospective study

Figure 3 presents the summary of this prospective study.

## Discussion

In this prospective study of children, adolescents and adults with T1D in Thailand, we found that poor sleep quality is common. Those with poor sleep quality were older but otherwise there were no other significant differences, including glycemic control, as compared to those with good sleep quality. Poor sleep quality, however, emerged as a predictor of severe hypoglycemia during comprehensive diabetes care without a relationship to glycemic control or DKA incidence. Each point increase in the PSQI score was associated with a 31% increase in the risk of severe hypoglycemia during follow up. These findings highlight the importance of sleep quality in T1D, particularly in relation to hypoglycemia risk.

The prevalence of poor sleep quality in this study of T1D is similar to previous reports, suggesting that this challenge is faced by T1D population among different regions of the world. A multicenter study in France of 315 adults with T1D (mean age 47 years) revealed that 59.8% reported poor sleep quality as assessed by PSQI [3]. A similar prevalence was found in the T1D Exchange cohort in the US of 515 children (age 2–12 years) [20], while the prevalence was 26% among 120 US adolescents who were not meeting their glycemic goal [4]. Only few studies were conducted outside Europe and North America. For example, a small study in Brazil found that 39% of T1D adults (mean age 26 years) reported excessive daytime sleepiness, reflecting a possibility of poor sleep quality [21]. In Japan, a survey of 192 T1D adults (mean age 55 years) revealed that around 75% had poor sleep quality as assessed by PSQI [22]. Meanwhile, our two previous small studies in Thailand (adults and adolescents) reported poor sleep quality in 40% of participants. Whether the prevalence of poor sleep quality in T1D is greater than that of the general population is debatable. While a meta-analysis revealed higher PSQI scores in T1D than in general population, the prevalence of poor sleep quality was similar [5]. Nevertheless, these data suggest that poor sleep quality is prevalent in T1D across age groups and regions worldwide.

Our study identified poor sleep quality as a predictor of severe hypoglycemia in participants receiving comprehensive diabetes treatment program including DSMES and free glucose testing supplies. Studies have revealed the relationship between poor sleep quality and hypoglycemia in T1D, although most were cross-sectional in nature [5, 23]. Hypoglycemia, particularly nocturnal hypoglycemia, is essentially experienced by all persons with T1D and can disrupt sleep quality [4]. Nocturnal hypoglycemia can lead to alterations of sleep stages and increased arousals [9]. Waking up to manage hypoglycemic events was common and patients reported difficulty returning back to sleep [23], further worsening sleep quality [24]. Fear of hypoglycemia could lead to anxiety which can disrupt sleep [3]. Poor sleep quality in T1D, regardless of the cause, has been shown to affect next day functioning, stress and self-management skills, potentially increasing hypoglycemic risks [25]. Therefore, the relationship between poor sleep and hypoglycemia in T1D appears to be bidirectional and potentially creating a vicious cycle. Our results confirm this relationship and further extend the knowledge of this relationship in a longitudinal context, suggesting that addressing sleep quality should be routinely incorporated when delivering DSMES in T1D.

Studies focusing on sleep interventions in T1D have recently emerged. Two studies in the US in teens [26] and school-aged children and their parents [27] utilized three sessions of sleep coaching. Among teens, there was an improvement in objectively measured sleep duration and subjective sleep quality, but no effects were found on diabetes self-care behaviors or A1C after 3 months [26]. Among children, sleep or diabetes outcomes did not change but parental sleep quality and well-being improved [27]. Another pilot randomized study in US adults revealed that an 8-week, remotely delivered intervention (consisting of digital lessons, sleep tracker, and weekly coaching phone calls) resulted in an improvement in sleep regularity, glycemic variability, and improved time in range as assessed by CGM in the intervention as compared to the control groups [28]. Fatigue and depressive mood also improved compared to the control [28]. While the results from these pilot studies are encouraging, larger scale randomized studies are needed to establish the benefits of sleep interventions in T1D.

Our study’s strength lies in its longitudinal design, but several limitations and differences from previous studies should be noted. We did not find a relationship between sleep and glycemic control as previously reported [5], possibly our relatively high mean A1C. While poor sleep quality was related to incident hypoglycemia possibly due to reduced self-care, SMBG frequencies were similar between those with poor vs. good sleep quality in the current study. Other aspects of self-care, such as insulin adherence, insulin dose calculation, diet and physical activity levels were not collected and could potentially be mediators between sleep quality and hypoglycemia. Furthermore, other relevant psychological measures, including depressive or anxiety symptoms which could relate to both hypoglycemia and sleep quality in T1D, were not assessed. It should be noted that our participants were not using diabetes technologies (e.g., AID, CGM) due to the limited availability in the country. The effects of such technologies on sleep have been mixed [29], though they have been associated with reduced hypoglycemia in a previous study [30]. Therefore, our results may not be generalizable to T1D populations with high utilities of these technologies. Moreover, sleep was assessed subjectively; objective measures such as actigraphy or polysomnography should be incorporated in future studies. Lastly, despite the longitudinal nature of the study, the causality between poor sleep quality and incident hypoglycemia could not be firmly established. Further research exploring the effects of sleep interventions in T1D and hypoglycemia, taking into account diabetes treatment modalities and other psychological factors, should be considered. Strategies to incorporate these interventions into routine care should also be explored.

In conclusion, this prospective study of T1D individuals in Thailand undergoing comprehensive diabetes care found that poor sleep quality is common and is a risk factor for incident severe hypoglycemia during a 12-month follow up. Each one-point increase in the PSQI score was associated with a 31% increased risk of severe hypoglycemia during follow-up. These findings underscore the importance of incorporating sleep assessment into routine T1D diabetes care and highlights the need of targeted interventions to improve sleep quality in individuals with T1D.

## Data Availability

The datasets generated during and/or analyzed during the current study are available from the corresponding author upon reasonable request.
